# Effect of diagnostic labelling on management intentions for non‐specific low back pain: A randomized scenario‐based experiment

**DOI:** 10.1002/ejp.1981

**Published:** 2022-06-21

**Authors:** Mary O'Keeffe, Giovanni E. Ferreira, Ian A. Harris, Ben Darlow, Rachelle Buchbinder, Adrian C. Traeger, Joshua R. Zadro, Rob D. Herbert, Rae Thomas, Joletta Belton, Chris G. Maher

**Affiliations:** ^1^ Institute for Musculoskeletal Health Sydney Local Health District and The University of Sydney Sydney New South Wales Australia; ^2^ Whitlam Orthopaedic Research Centre Ingham Institute for Applied Medical Research, South Western Sydney Clinical School, The University of New South Wales Sydney New South Wales Australia; ^3^ Department of Primary Healthcare and General Practice University of Otago Wellington New Zealand; ^4^ Monash Department of Clinical Epidemiology Cabrini Institute Melbourne Australia; ^5^ Department of Epidemiology and Preventive Medicine School of Public Health and Preventive Medicine, Monash University Melbourne Australia; ^6^ Neuroscience Research Australia (NeuRA) Randwick New South Wales Australia; ^7^ University of New South Wales Randwick New South Wales Australia; ^8^ Faculty of Health Sciences and Medicine Institute for Evidence‐Based Healthcare, Bond University Gold Coast Queensland Australia; ^9^ Endless Possibilities Initiative Fraser Colorado USA

## Abstract

**Background:**

Diagnostic labels may influence treatment intentions. We examined the effect of labelling low back pain (LBP) on beliefs about imaging, surgery, second opinion, seriousness, recovery, work, and physical activities.

**Methods:**

Six‐arm online randomized experiment with blinded participants with and without LBP. Participants received one of six labels: **‘** *disc bulge’,* **‘** *degeneration’,* **‘** *arthritis’,* **‘** *lumbar sprain’,* **‘** *non‐specific LBP’*, **‘** *episode of back pain’*. The primary outcome was the belief about the need for imaging.

**Results:**

A total of 1375 participants (mean [SD] age, 41.7 years [18.4 years]; 748 women [54.4%]) were included. The need for imaging was rated lower with the labels **‘** *episode of back pain’* (4.2 [2.9]), **‘** *lumbar sprain’* (4.2 [2.9]) and **‘** *non‐specific LBP’* (4.4 [3.0]) compared to the labels **‘** *arthritis’* (6.0 [2.9]), **‘** *degeneration’* (5.7 [3.2]) and **‘** *disc bulge’* (5.7 [3.1]). The same labels led to higher recovery expectations and lower ratings of need for a second opinion, surgery and perceived seriousness compared to **‘** *disc bulge’,* **‘** *degeneration’* and **‘** *arthritis’*. Differences were larger amongst participants with current LBP who had a history of seeking care. No differences were found in beliefs about physical activity and work between the six labels.

**Conclusions:**

**‘** *Episode of back pain’,* **‘** *lumbar sprain’* and **‘** *non‐specific LBP’* reduced need for imaging, surgery and second opinion compared to **‘** *arthritis’,* **‘** *degeneration’* and **‘** *disc bulge’* amongst public and patients with LBP as well as reducing the perceived seriousness of LBP and enhancing recovery expectations. The impact of labels appears most relevant amongst those at risk of poor outcomes (participants with current LBP who had a history of seeking care).

## 1 INTRODUCTION

Low back pain (LBP) is the leading cause of years lived with disability worldwide (Vos et al., 2015). It is the second most common symptom‐related reason for seeking care from a primary care provider (Deyo & Weinstein, 2001). In 2016, in the United States, an estimated $134.5 billion was spent on health services for patients with low back and neck pain (ranked first amongst 154 health conditions), and this spending is rapidly increasing each year (Dieleman et al., 2020). Non‐specific LBP is the guideline‐recommended label for the vast majority (90%–95%; Deyo & Weinstein, 2001) of LBP. This refers to LBP where it is currently not possible to identify a specific structural cause (e.g. radiculopathy, fracture, malignancy; Bardin et al., 2017; Maher et al., 2017).

The view that we cannot identify the cause of most LBP is an unpopular one, (Bishop et al., 2015; Bogduk, 2000; Kent & Keating, 2004) and so the non‐specific LBP label receives heavy criticism. Opponents of the non‐specific label claim it is cumbersome to use with patients; conveys that the clinician does not know what is wrong with the patient; provides no pathoanatomical basis for LBP, and is a barrier to the provision of individualized care (Bishop et al., 2015; Bogduk, 2000; Kent & Keating, 2004). In fact, The North American Spine Society (the largest medical spine society in the United States), in their 2020 clinical guideline for the diagnosis and assessment of LBP, appeared to reject the non‐specific LBP label; ‘The term “non specific LBP” provides no biologic basis for LBP nor assistance in clinical decision‐making’ and ‘further studies of non‐specific LBP are unwarranted’ (North American Spine Society, 2020).

Clinicians commonly use other labels to describe LBP not linked to a specific structural cause. For example, a survey study of 1093 primary‐contact clinicians found that 74% think it is possible to identify the source in all cases of LBP and that clinicians treat differently based on patterns of signs and symptoms of presumed structural sources of LBP, including intervertebral discs, facet joints, lumbar ligaments and lumbar muscles (Kent & Keating, 2004). Diagnostic labels signifying pathology relating to these structures are used in clinical practice. These include ‘disc bulge’, ‘degeneration’, ‘arthritis’ and ‘lumbar sprain’. All feature prominently in disease classification systems, including the International Classification of Diseases. Like non‐specific LBP, the use of these specific structural labels is considered problematic for three reasons: (1) The clinical tests used to identify potential structural sources of LBP (e.g. disc degeneration) have low validity (Hancock et al., 2007). (2) The actual clinical importance of these structural findings is debatable. For example, a systematic review (33 studies, 3310 asymptomatic individuals) concluded that the prevalence of disc bulge was 30% in 20‐year‐olds, 60% in 50‐year‐olds and increased to 84% in 80‐year‐olds amongst asymptomatic individuals, whilst the prevalence of disc degeneration amongst asymptomatic individuals increased from 37% in 20‐year‐olds to 90% in 80‐year‐olds (Brinjikji et al., 2015). (3) Some structural labels may carry negative connotations, and influence recovery expectations and beliefs about work and physical activity. For example, the label ‘degeneration’ may convey to a patient that their back is fragile (Bogduk, 2000).

Diagnostic labels may be important as patients want an explanation for their LBP (Bogduk, 2000; Jenkins et al., 2016). However, concerns have been expressed that clinicians may lack an adequate vocabulary for explaining LBP not linked to a specific structural cause (Bogduk, 2000). It is unclear whether current labels used for this form of LBP reassure patients that their LBP is not dangerous, or improve the expectation of a positive outcome. Certain labels could trigger ‘therapeutic misadventure’ (Bogduk, 2000). For example, some labels (e.g. disc degeneration) may have the potential to influence patients' desire to get unnecessary lumbar imaging. In fact, clinicians often report that patient desire is a key driver of imaging behaviour (Slade et al., 2015; Slade et al., 2016). Unnecessary imaging can cause harm. Misinterpretation of imaging results by clinicians could result in unhelpful advice (e.g. staying off work) and a cascade of medical interventions (Lemmers et al., 2019; Webster et al., 2013; Webster et al., 2014). For example, asymptomatic disc degeneration is common and so unnecessary imaging could trigger overdiagnosis and the overuse of ineffective and costly treatments (e.g. lumbar fusion surgery).

Potential negative impacts of some labels (e.g. disc degeneration) for LBP have been suggested in some qualitative and retrospective cohort studies (Abenhaim et al., 1995; Darlow et al., 2013; Darlow et al., 2015; Sloan & Walsh, 2010). However, the impact of different diagnostic labels used for LBP on patients has not been explored through rigorous experimental studies. Consequently, there is no robust evidence to guide clinicians' use of different labels. We, therefore, investigated the effects of diagnostic labels for LBP on patients' perceived need for imaging. Secondary aims were to evaluate the effects of labelling on willingness to undergo surgery, beliefs about the need for a second opinion, perceived seriousness of LBP, recovery expectations and beliefs about the ability to engage with work and physical activities.

## 2 METHODS

### 2.1 Study design

This was a six‐arm, parallel group, superiority randomized experiment with blinded participants conducted online. The study was approved by The University of Sydney Human Research Ethics Committee (2019/539).

### 2.2 Participants

Participants were recruited through Qualtrics (www.qualtrics.com). Qualtrics uses existing, nationally representative panels of individuals who have previously agreed to participate in research. We recruited three groups of participants: (1) Adults who have LBP and have received formal treatment for LBP at any time in their life (e.g. treatment from a doctor, physical therapist, chiropractor, surgeon, or any other healthcare provider). (2) Adults who have LBP and have never received formal treatment for LBP. (3) Adults who have never experienced LBP in their lifetime. We stopped recruitment within each group when it reached the target number of participants. We defined an episode of LBP as pain lasting for at least 24 h. We assessed LBP in the past week using the 0–10 Numeric Rating Scale (Jensen et al., 1999). Included participants were 18 years or older, able to read and write English, and living in Australia, Canada or Ireland. We picked these three countries due to having similar healthcare models. There were no other restrictions to participation. All participants completed an online consent form after reading the participant information sheet.

### 2.3 Procedure

All participants were provided the same scenario (Box 1) of attending a primary care clinician about LBP. The scenario described the location of the pain, possible triggering event, and functional limitations. Participants were then randomized to receive one of six diagnostic labels with explanations: ‘you have a *disc bulge*’; ‘you have *degeneration* of the spine’; ‘you have *arthritis* of the spine’; ‘you have a *lumbar sprain*’; ‘you have *non‐specific low back pain* ’or’ you have an *episode of back pain*’, using the web‐based, block randomization allocation system provided by Qualtrics.

BOX 1 **Low back pain scenario**

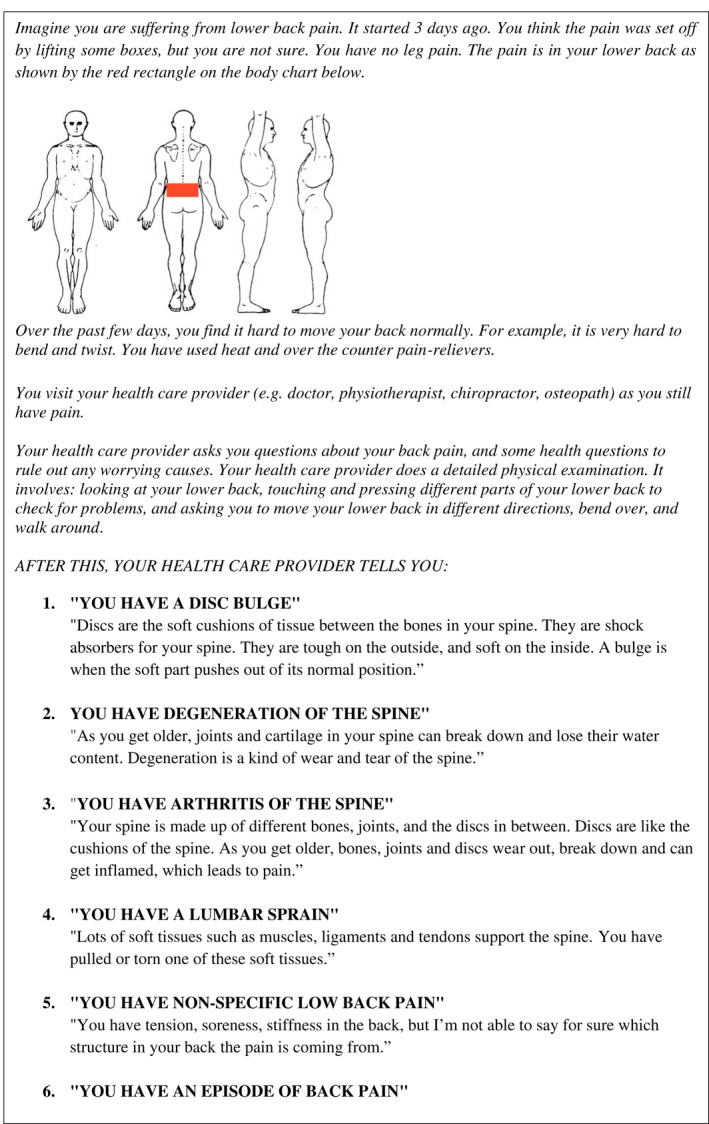

All six groups then received the same reassurance from the primary care clinician: ‘I'm not worried that there is anything serious going on here. I think overall your outlook is good. Movement will help. The sooner we can get you back to your normal activity and work, the more likely your back pain is to get better’.

We chose to test *disc bulge*, *degeneration* and *arthritis* as they are common imaging findings in asymptomatic individuals and may be of doubtful clinical significance. They are also mentioned in the qualitative literature as carrying potentially negative connotations, and they are included in medical disease classification systems. We chose *lumbar sprain* as it is included in medical disease classification systems, it is commonly used by clinicians for LBP with no specific structural cause, (Darlow et al., 2014) and that patients perceive this diagnosis as an injury (Darlow et al., 2013). We chose *non‐specific LBP* as it is the guideline‐recommended term for most LBP, but seems unpopular amongst many clinicians. We chose an *episode of back pain* to describe the symptom of LBP without attaching any structural descriptor.

### 2.4 Outcome measures

#### 2.4.1 Primary outcome

The primary outcome was a belief about the need for imaging for LBP. This was assessed using a single item on an 11‐point Likert scale (0 = definitely not; 10 = definitely do), adapted from previous research (Fisher et al., 2012). *Do you think you need a scan (for example, an X‐ray or MRI scan) of your back?*

#### 2.4.2 Secondary outcomes

Willingness to undergo surgery for LBP, belief in need for a second opinion for LBP, perceived seriousness of LBP and recovery expectations, were each assessed with a single question on an 11‐point Likert scale, adapted from previous research (Fisher et al., 2012; Hallegraeff et al., 2012). Additional secondary outcomes included beliefs about physical activity and beliefs about work, assessed by two 7‐point Likert‐type questions from the Fear Avoidance Beliefs Questionnaire (Waddell et al., 1993). Details on the anchoring wording for each of the outcomes are described in eMethods in the Supplement.

We collected a range of demographic and healthcare utilization data. These data included age, gender, educational attainment, employment status, history of diagnostic investigations for LBP (X‐ray, MRI), history of lumbar surgery, history of sick leave due to LBP, history of receiving a diagnosis for LBP, pain intensity (Numerical rating scale [0–10]) and functional disability (Oswestry Disability Index [0–100]), duration of LBP, back beliefs, anxiety and depression. Details on the specific questionnaires used are described in eMethods in the Supplement.

### 2.5 Sample size

A power calculation conducted using a simulation approach (Landau & Stahl, 2013) indicated that 1296 participants were required to have an 80% power to detect a difference of 1 point (difference chosen by the author team) in one of the six labels for belief about the need for imaging (primary outcome), assuming a standard deviation of 3 and a correlation between previous imaging and outcome of 0.3. Qualtrics pilot tested our trial on a group of 175 participants prior to recruitment so we could perform data checks and correct typos.

### 2.6 Statistical analysis

Descriptive statistics (means and standard deviations [SD], counts, and percentages) were used to summarize demographic, healthcare utilization and outcome data across the six groups. Differences in means between the groups were compared using analysis of covariance for all outcomes. To control the family‐wise Type I error rate, the Bonferroni correction was used. As such, between‐group differences were declared significant at the level of *p* < 0.0033 (two‐tailed hypothesis) and we calculated 99.67% confidence intervals (CI). For the primary outcome, we adjusted for previous imaging for LBP (yes/no) measured at baseline. For the willingness to undergo surgery, we adjusted for previous surgery for LBP (yes/no) measured at baseline. The remainder of the outcomes were left unadjusted. We performed a subgroup analysis on all outcomes to examine if the effect of labelling varied across the three groups of participants (no history of LBP, current LBP ([history of seeking care], current LBP [no history of seeking care]). Participants who did not complete a primary or secondary outcome were excluded from all analyses. Analyses were performed using Stata, version 16.0 (StataCorp LLC).

## 3 RESULTS

**FIGURE 1:**
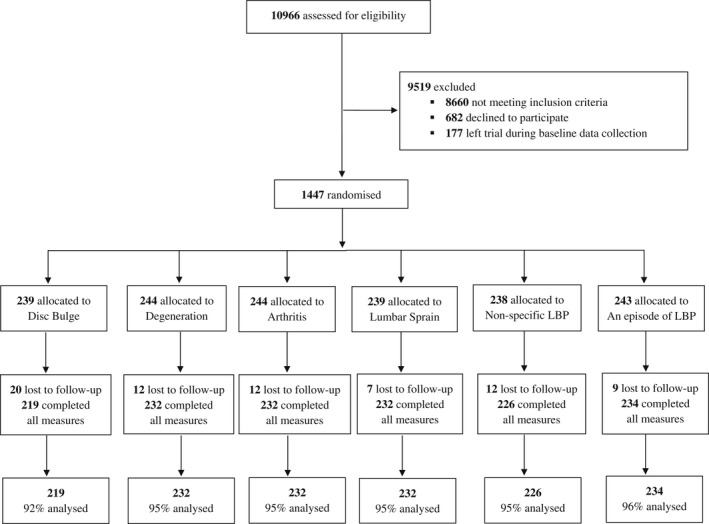
Flowchart of participants in the trial of labelling for LBP

Recruitment and data collection took place from 12 October 2019 to 6 December 2019. Of the 10,966 individuals assessed for eligibility, 1447 were randomized. A total of 72 participants (5%) did not complete outcome measures leaving 1375 participants (95% compliance rate) who were included in our analyses (Figure 1). We experienced difficulty recruiting participants with no history of LBP and we continued to assess people for eligibility to join this group after recruitment had concluded for the other two participant groups. A large number of individuals (n = 8860) were not eligible to join our no history of LBP group.

Baseline characteristics (e.g. age, sex, back beliefs, depression, anxiety, healthcare utilization for LBP, etc.) were similar between the six randomized groups. Participants were on average 41.7 years old and 54.4% were female. Participants with current LBP had a mean pain intensity of 5.1/10 (SD 2.3) and a mean disability of 17.6/100 (SD 13.6). Most participants with current LBP (37.9%) had LBP for greater than 12 weeks. A similar proportion of participants with current LBP had previously received a diagnostic label for LBP (27.9%) and had a history of imaging (24.7%). A smaller proportion of participants with current LBP had received surgery for LBP (3.6%) (Table 1).

**TABLE 1:** Baseline characteristics of participants

	Total sample (*n* = 1375)	Disc bulge (*n* = 219)	Degeneration (*n* = 232)	Arthritis (*n* = 232)	Lumbar sprain (*n* = 232)	Non‐specific LBP (*n* = 226)	Episode of LBP (*n* = 234)
Type of participant, *n* (%)							
No history of LBP	359 (26.1)	50 (22.8)	63 (27.2)	62 (26.7)	62 (26.7)	57 (25.2)	65 (27.7)
Current LBP (history of seeking care)	507 (36.9)	84 (38.4)	85 (36.6)	85 (36.6)	85 (36.6)	84 (37.2)	84 (35.9)
Current LBP (no history of seeking care)	509 (37.0)	85 (38.8)	84 (36.2)	85 (36.6)	85 (36.6)	85 (37.6)	85 (36.3)
Age (years), mean (SD)	41.7 (18.4)	41.9 (17.6)	42.1 (19.4)	41.7 (19.1)	42.7 (18.6)	41.8 (18.0)	39.9 (17.9)
Female, *n* (%)	748 (54.4)	125 (57.1)	119 (51.3)	138 (59.5)	132 (56.9)	117 (51.8)	117 (50.0)
Pain intensity (0–10), mean (SD)	3.8 (3.0)	3.9 (2.9)	3.8 (3.1)	3.8 (2.9)	3.7 (2.9)	3.8 (2.9)	3.8 (3.1)
*Participants with LBP*	5.1 (2.3)	5.0 (2.3)	5.2 (2.4)	5.2 (2.1)	5.1 (2.2)	5.0 (2.3)	5.3 (2.3)
Disability (0–100), (SD)	13.0 (14.0)	12.3 (14.0)	14.2 (15.3)	13.3 (14.6)	12.7 (13.3)	13.0 (13.2)	12.7 (13.7)
*Participants with LBP*	17.6 (13.6)	15.9 (14.1)	19.5 (14.8)	18.1 (14.3)	17.4 (12.6)	17.4 (12.5)	17.6 (13.2)
Previous imaging for LBP (yes), (%)	340 (24.7)	60 (27.4)	57 (24.6)	57 (24.6)	48 (20.7)	60 (26.6)	58 (24.8)
Previous surgery for LBP (yes), (%)	50 (3.6)	7 (3.2)	12 (5.2)	8 (3.5)	8 (3.5)	7 (3.1)	8 (3.4)
Previous sick leave for LBP (yes), *n* (%)	346 (25.2)	54 (24.7)	56 (24.1)	56 (24.1)	58 (25.0)	56 (24.8)	66 (28.2)
Previous LBP diagnosis given (yes), *n* (%)	384 (27.9)	62 (28.3)	57 (24.6)	68 (29.3)	67 (28.9)	60 (26.6)	70 (29.9)
Education, *n* (%)							
High school (not completed)	109 (7.9)	14 (6.4)	15 (6.5)	23 (9.9)	22 (9.5)	21 (9.3)	14 (6.0)
High school (completed)	337 (24.5)	61 (27.9)	65 (28.0)	55 (23.7)	48 (20.7)	47 (20.8)	61 (26.1)
TAFE/Trade	323 (23.5)	49 (22.4)	51 (21.9)	59 (25.4)	61 (26.3)	54 (23.9)	49 (20.9)
University	606 (44.1)	95 (43.4)	101 (43.6)	95 (40.9)	101 (43.5)	104 (46.0)	110 (47.0)
Employment, *n* (%)							
Employed	943 (68.6)	160 (73.1)	153 (65.9)	153 (65.9)	155 (66.8)	160 (70.8)	162 (69.2)
Unemployed	149 (10.9)	25 (11.4)	21 (9.1)	26 (11.2)	29 (12.5)	27 (11.9)	21 (9.0)
Student	87 (6.3)	13 (5.9)	16 (6.9)	16 (6.9)	13 (5.6)	10 (4.3)	19 (8.2)
Retired	196 (14.3)	21 (9.6)	42 (18.1)	37 (15.9)	35 (15.1)	29 (12.8)	32 (13.7)
Duration of current LBP, *n* (%)							
Not applicable (No LBP)	359 (26.1)	50 (22.8)	63 (27.2)	62 (26.7)	62 (26.7)	57 (25.2)	65 (27.8)
Less than 1 week	251 (18.3)	57 (26.0)	36 (15.5)	38 (16.4)	38 (16.4)	40 (17.7)	42 (17.9)
1–6 weeks	173 (12.6)	23 (10.5)	32 (13.8)	34 (14.7)	29 (12.5)	28 (12.4)	27 (11.5)
7–12 weeks	70 (5.1)	12 (5.5)	12 (5.2)	12 (5.2)	7 (3.0)	10 (4.4)	17 (7.3)
Longer than 12 weeks	522 (37.9)	77 (35.2)	89 (38.4)	86 (37.1)	96 (41.4)	91 (40.3)	83 (35.5)
General health, *n* (%)							
Very good	243 (17.7)	34 (15.5)	37 (15.9)	42 (18.1)	38 (16.4)	38 (16.8)	54 (23.1)
Good	735 (53.5)	128 (58.5)	130 (56.0)	119 (51.3)	116 (50.0)	122 (53.9)	120 (51.3)
Neither good nor poor	287 (20.9)	41 (18.7)	40 (17.2)	53 (22.8)	57 (24.6)	49 (21.7)	47 (20.1)
Poor	95 (6.9)	16 (7.3)	22 (9.5)	12 (5.2)	18 (7.8)	15 (6.6)	12 (5.1)
Very poor	15 (1.1)	0 (0)	3 (1.3)	6 (2.6)	3 (1.3)	2 (0.9)	1 (0.4)
Anxiety (0–10), mean (SD)	4.5 (2.9)	4.5 (2.9)	4.3 (3.0)	4.7 (2.9)	4.6 (2.8)	4.6 (3.0)	4.3 (3.0)
Depression (0–10), mean (SD)	3.7 (3.1)	3.6 (3.1)	3.6 (3.1)	4.0 (3.2)	3.8 (2.9)	3.8 (3.1)	3.34 (3.1)
Back Beliefs Questionnaire (9–45, higher score indicated better beliefs), mean (SD)	29.3 (4.9)	29.1 (5.02)	29.1 (5.3)	29.5 (4.9)	29.4 (4.7)	29.1 (4.5)	29.6 (5.3)

### 3.1 Primary outcome

#### 3.1.1 Need for lumbar imaging (0–10)

**FIGURE 2:**
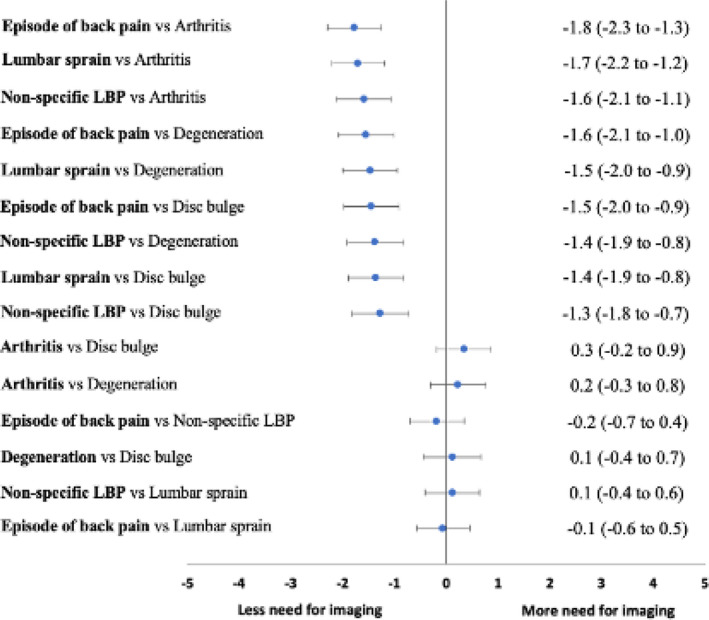
Adjusted mean differences (99.67% CIs) for beliefs about the need for imaging on an 11‐point Likert scale.

Participants who received the labels *episode of back pain* (mean [SD] 4.2 [2.9]), *lumbar sprain* (4.2 [2.9]) and *non‐specific LBP* (4.4 [3.0]) perceived less need for lumbar imaging compared to those receiving the labels *arthritis* (6.0 [2.9]), *degeneration* (5.7 [3.2]) and *disc bulge* (5.7 [3.1]) An *episode of back pain* consistently had the lowest perceived need for imaging in comparison to *arthritis*, *degeneration* and *disc bulge*, followed by *lumbar sprain* and *non‐specific LBP* (Table 2 and Figure 2).

**TABLE 2:** Primary and secondary outcomes stratified by our three groups of participants

	Participant group	Disc bulge	Degeneration	Arthritis	Lumbar sprain	Non‐specific LBP	Episode of back pain
Mean (SD)							
Primary Outcome							
Imaging (0–10)^a^	No history of LBP	5.6 (3.1)	4.9 (3.4)	5.2 (3.1)	4.0 (2.7)	4.2 (3.1)	4.0 (2.9)
	Current LBP (history of care seeking)	6.3 (2.8)	6.6 (2.6)	6.5 (2.7)	4.8 (3.1)	5.0 (2.9)	4.2 (2.8)
	Current LBP (no history of care seeking)	5.0 (3.2)	5.5 (3.3)	5.9 (2.8)	3.7 (2.7)	3.8 (2.8)	4.3 (3.0)
Secondary outcomes							
Second opinion (0–10)	No history of LBP	4.4 (2.7)	4.9 (3.3)	5.2 (3.2)	3.5 (2.6)	4.4 (3.3)	4.6 (3.1)
	Current LBP (history of care seeking)	5.4 (2.7)	6.3 (2.6)	5.9 (3.1)	4.0 (2.7)	5.3 (2.9)	4.4 (2.8)
	Current LBP (no history of care seeking)	5.2 (3.0)	5.3 (3.0)	5.8 2.6)	3.8 (2.7)	3.9 (3.0)	4.6 (3.1)
Willing to undergo surgery (0–10)^b^	No history of LBP	4.6 (2.9)	4.8 (3.2)	4.1 (2.9)	3.8 (2.8)	3.9 (3.0)	3.7 (2.8)
	Current LBP (history of care seeking)	4.5 (3.0)	4.6 (2.9)	4.4 (3.1)	3.6 (3.1)	3.7 (2.9)	3.6 (2.9)
	Current LBP (no history of care seeking)	3.9 (2.8)	4.5 (2.9)	3.9 (2.7)	3.5 (2.7)	2.7 (2.5)	3.7 (2.9)
Perceived seriousness (0–10)	No history of LBP	6.1 (2.0)	6.1 (2.6)	6.0 (2.5)	3.9 (2.5)	4.1(2.7)	4.4 (2.9)
	Current LBP (history of care seeking)	6.1 (2.4)	7.0 (1.9)	6.8 (2.3)	4.7 (2.6)	4.6 (2.4)	5.0 (2.5)
	Current LBP (no history of care seeking)	5.4 (2.6)	6.5 (2.4)	6.0 (2.3)	4.0 (2.4)	3.7 (2.4)	4.1 (2.7)
Recovery expectations(0–10)	No history of LBP	5.6 (2.3)	4.7 (2.4)	4.2 (2.7)	7.1 (2.4)	6.0 (2.7)	5.4 (2.7)
	Current LBP (history of care seeking)	5.4 (2.5)	4.5 (2.6)	4.4 (2.6)	6.6 (2.4)	5.4 (2.7)	6.1 (2.6)
	Current LBP (no history of care seeking)	5.6 (2.4)	4.8 (2.4)	4.7 (2.4)	6.2 (2.7)	5.8 (2.7)	6.3 (2.6)
Physical activity may harm my back (0–6)	No history of LBP	3.7 (1.4)	3.3 (1.9)	3.3 (1.6)	3.2 (1.4)	3.3 (1.7)	3.1 (1.8)
	Current LBP (history of care seeking)	3.9 (1.5)	3.8 (1.4)	3.5 (1.6)	3.6 (1.5)	3.6 (1.4)	3.7 (1.4)
	Current LBP (no history of care seeking)	3.6 (1.5)	3.4 (1.6)	3.5 (1.5)	3.2 (1.6)	3.5 (1.4)	3.4 (1.6)
I should not do physical activities … (0–6)	No history of LBP	3.8 (1.5)	2.9 (1.9)	3.4 (1.7)	3.6 (1.8)	3.2 (1.7)	3.2 (1.9)
	Current LBP (history of care seeking)	3.7 (1.8)	3.8 (1.5)	3.4 (1.6)	3.6 (1.8)	3.5 (1.6)	3.6 (1.4)
	Current LBP (no history of care seeking)	3.5 (1.7)	3.2 (1.7)	3.3 (1.5)	3.2 (1.8)	3.3 (1.6)	3.7 (1.7)
My work might harm my back(0–6)	No history of LBP	3.1 (1.5)	2.9 (1.6)	2.8 (1.6)	2.9 (1.5)	2.9 (1.5)	2.9 (1.7)
	Current LBP (history of care seeking)	3.3 (1.6)	3.5 (1.7)	3.2 (1.7)	3.4 (1.7)	3.4 (1.6)	3.2 (1.5)
	Current LBP (no history of care seeking)	2.9 (1.6)	2.7 (1.6)	2.8 (1.4)	2.6 (1.6)	2.8 (1.6)	3.0 (1.4)
I should not do my normal work (0–6)	No history of LBP	3.3 (1.6)	3.2 (1.8)	2.8 (1.6)	2.9 (1.5)	2.7 (1.6)	2.9 (1.6)
	Current LBP (history of care seeking)	2.8 (1.6)	3.8 (1.6)	3.1 (1.6)	3.0 (1.7)	3.0 (1.6)	3.0 (1.6)
	Current LBP (no history of care seeking)	2.8 (1.7)	3.3. (1.6)	2.7 (1.3)	2.6 (1.7)	2.2 (1.5)	2.6 (1.7)

^a^ Adjusted for previous lumbar imaging.

^b^ Adjusted for previous surgery for LBP.

These differences between labels were evident across our three groups of participants. However, there were larger differences for perceived need for imaging between the labels for participants with current LBP who had a history of seeking care (Table 2).

### 3.2 Secondary outcomes

#### 3.2.1 Willingness to undergo surgery (0–10)

**FIGURE 3:**
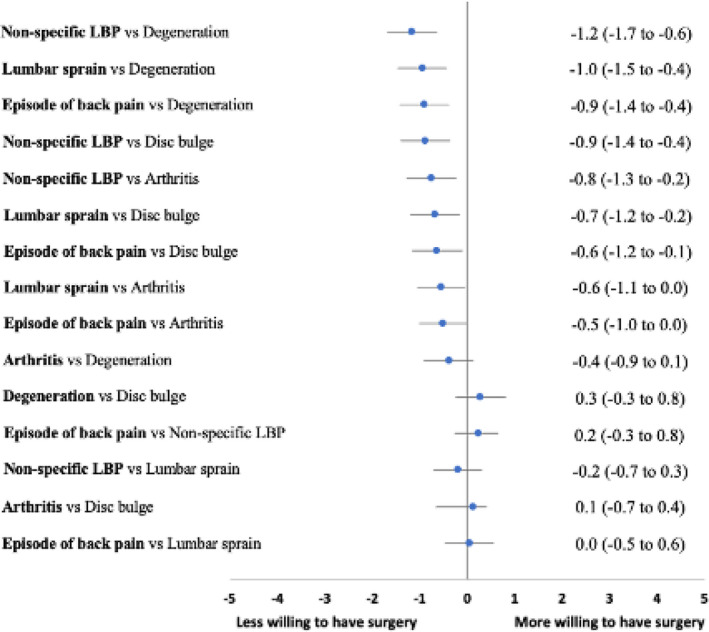
Adjusted mean differences (99.67% CIs) for willingness to undergo surgery on an 11‐point Likert scale.

Participants who received the labels *non‐specific LBP* (3.4 [2.8]), *lumbar sprain* (3.6 [2.9]) and *episode of back pain* (3.7 [2.9]) were less willing to undergo surgery compared to those receiving the labels *degeneration* (4.6 [3.0]), *disc bulge* (4.3 [2.9]), and *arthritis* (4.2 [2.9]). *Non‐specific LBP* consistently had the lowest perceived need for surgery in comparison to *degeneration*, *disc bulge* and *arthritis*, followed by *lumbar sprain* and *episode of back pain* (Table 2 and Figure 3).

These differences between labels were evident across our three groups of participants. However, there were larger differences for perceived need for surgery between the labels for participants with current LBP who had a history of seeking care (Table 2).

#### 3.2.2 Need for a second opinion (0–10)

Participants who received the labels *lumbar sprain* (3.6 [2.9]), *episode of back pain* (4.6 [3.0]) and *non‐specific LBP* (4.6 [3.1]) perceived less need for a second opinion compared to those receiving the labels *arthritis* (5.7 [3.0]), *degeneration* (5.6 [3.0]) and *disc bulge* (5.1 [2.9]). *Lumbar sprain* consistently had the lowest perceived need for a second opinion compared to the other five labels. Participants who received the label *arthritis* perceived a higher need for a second opinion compared to those receiving the label *disc bulge* (mean difference [MD] 0.6, 99.67% CI: 0.1–1.2) (Table 2 and eFigure 1 in the Supplement).

These differences between labels were evident across our three groups of participants. However, there were larger differences for perceived need for a second opinion between the labels for participants with current LBP who had a history of seeking care (Table 2).

#### 3.2.3 Perceived seriousness of LBP (0–10)

Participants who received the labels *non‐specific LBP* (4.1 [2.5]), *lumbar sprain* (4.2 [2.6]) and *episode of back pain* (4.5 [2.7]) perceived LBP as less serious compared to those receiving the labels *degeneration* (6.6 [2.3]), *arthritis* (6.3 [2.4]) and *disc bulge* (5.9 [2.4]). Participants who received the label *disc bulge* perceived LBP as less serious compared to those receiving *degeneration. Non‐specific LBP* had the lowest perceived seriousness of LBP in comparison to *degeneration*, arthritis and *disc bulge*, followed by *lumbar sprain* and *episode of back pain* (Table 2 and eFigure 2 in the Supplement).

These differences between labels were evident across our three groups of participants. However, there were larger differences for perceived seriousness of LBP between the labels for participants with current LBP who had a history of seeking care (Table 2).

#### 3.2.4 Recovery expectations (0–10)

Participants who received the labels *lumbar sprain* (6.6 [2.4]), *episode of back pain* (6.0 [2.6]) and *non‐specific LBP* (5.7 [2.7]) had higher recovery expectations compared to those receiving the labels *arthritis* (4.4 [2.5]), *degeneration* (4.7 [2.5]) and *disc bulge* (5.5 [2.4]). Participants who received the labels *arthritis* and *degeneration* had lower recovery expectations compared to those receiving *disc bulge. Lumbar sprain* consistently had the highest perceived recovery expectations in comparison to *arthritis*, *degeneration*, *disc bulge* and *non‐specific LBP*, followed by *episode of LBP* (Table 2 and eFigure 3 in the Supplement).

These differences between labels were evident across our three groups of participants. However, there were larger differences in recovery expectations between the labels for participants with current LBP who had a history of seeking care (Table 2).

#### 3.2.5 Engaging in work and physical activities (0–6)

For beliefs about engaging in normal work with pain, participants who received the label *degeneration* (3.5 [1.7]) were more likely to agree that they should not work compared to participants who received the labels *disc bulge* (2.9 [1.6]), *arthritis* (2.9 [1.5]), *episode of back pain* (2.9 [1.7]), *lumbar sprain* (2.8 [1.7]) and *non‐specific LBP* (2.6 [1.6]). Apart from this, we found little to no differences in beliefs about physical activity and work being harmful between the six labels (Table 2 and eFigures 4–7 in the Supplement).

## 4 DISCUSSION

### 4.1 Summary of key findings

This randomized experiment provides evidence that the assignment of some diagnostic labels (*episode of back pain, lumbar sprain, non‐specific LBP*) reduced the perceived need for imaging, surgery and second opinion compared to other labels (*arthritis, degeneration and disc bulge*) amongst individuals with and without LBP. Assignment of the same labels (*lumbar sprain, non‐specific LBP* and *episode of back pain*) also reduced the perceived seriousness of LBP and increased recovery expectations. Importantly, the impact of labels appears most relevant amongst those at risk of poor outcome (participants with current LBP who had a history of seeking care), suggesting that what may be a benign label (e.g. disc bulge) amongst many might be dangerous/risky amongst the vulnerable. Interestingly, no difference was found in beliefs about physical activity and work being harmful between the six labels.

This experiment suggests that certain diagnostic labels (*arthritis*, *degeneration* and *disc bulge*) have the effect of encouraging tests (e.g. lumbar imaging) and treatments (e.g. surgery).

### 4.2 Comparison to the existing literature

To our knowledge, this is the first randomized study to examine the effect of diagnostic labels on beliefs and management preferences in the area of LBP. Our findings align with randomized trial evidence from other health areas (e.g. shoulder pain, cancer, conjunctivitis, polycystic ovary syndrome, gastroesophageal symptoms) that labels which medicalise a health condition or symptom increase intentions for more aggressive treatment options (Copp et al., 2017; McCaffery et al., 2015; Nickel et al., 2017; Scherer et al., 2013; Scherer et al., 2016; Zadro et al., 2021). More broadly, our findings support findings from qualitative research in the area of LBP that patients perceive labels such as disc bulge and degeneration as threatening and associated with poorer outcome (Darlow et al., 2013; Darlow et al., 2015; Sloan & Walsh, 2010).

### 4.3 Strengths and weaknesses of this study

Strengths of this study are the use of sound methods to reduce bias including randomization, concealed allocation, a sample size calculation and 99.67% confidence intervals to account for multiple analyses. To include a diversity of viewpoints, we included people with and without LBP with varying demographics (e.g. age, sex, work status) and experiences of healthcare utilization (e.g. previous imaging and lumbar surgery) for LBP. This is in contrast to many studies examining the effect of labelling on health intentions that include healthy people without the health condition of interest (Copp et al., 2017; McCaffery et al., 2015; Nickel et al., 2017; Scherer et al., 2013; Scherer et al., 2016). In addition, a consumer with experience of persistent non‐specific LBP helped co‐design the study and is a co‐author. We also recruited a large sample of participants to examine a variety of popular diagnostic labels for LBP and provided these labels along with guideline recommended reassurance and positive expectations of recovery.

The limitations of this study are that it was based on a scenario and results may differ in real‐world situations. However, pain intensity levels were similar to clinical populations. Online recruitment may select participants who are more technologically inclined; however, we recruited participants of varying ages and educational attainment. Outcome measurement was only at a single time point immediately after the labels were given; management preferences may change as participants reflect over time. The exclusion of missing outcome data may introduce bias. However, the missing proportion was low (<10%) and bias would be negligible. We did not prospectively register this study. We made this decision as it did not fit the WHO criteria for a clinical trial. However, we had a formal protocol, and the reporting of outcomes was in accordance with those plans.

### 4.4 Meaning of this study

Reducing the use of ineffective medical tests and treatments for non‐specific LBP is a research policy priority (Buchbinder et al., 2018; Foster et al., 2018). Diagnostic imaging (e.g. x‐ray and MRI) does not have a routine role in the management of non‐specific LBP (90%–95% (Deyo & Weinstein, 2001) of all LBP) (Maher et al., 2017). Yet about 25% of all patients who present to primary care with LBP are referred for imaging (Downie et al., 2019). Since clinician perceived patient expectations may have a large influence on clinician referral decisions, using labels that increase patients' perceived need for imaging could increase the actual amount of imaging received. Lumbar fusion surgery, a surgery commonly performed for a diagnosis of degeneration, provides no benefit over safer and less costly approaches such as exercise (Mannion et al., 2016) yet the US spends more money on spinal fusion each year than any other surgery (US$12.8 billion per annum) and it is the fourth amongst the surgeries generating the greatest cost in Australia (AUD650M per annum) (Maher et al., 2019). Clinical guidelines recommend advice and reassurance to help reduce or avoid unnecessary tests and treatments for non‐specific LBP. Consistent recommendations include educating people about the nature of LBP, reassurance that it is not a serious disease and will improve, and encouragement to avoid bed rest, stay active, and return to usual activities. Our study found that providing reassurance does not remove the negative effects of the labels *arthritis*, *degeneration* and *disc bulge*. Overall, this study suggests that clinicians could consider avoiding labels like *arthritis, degeneration* and *disc bulge*. Instead, clinicians could consider using labels like an *episode of back pain*, *lumbar sprain* or *non‐specific LBP* when communicating with patients with LBP, where any specific structural cause needing further exploration has been reasonably excluded. Removing labels like *degeneration* from low‐risk LBP presentations (i.e. non‐specific LBP) may help shift patients' perspectives and enable them to feel more comfortable with accepting a non‐medical treatment option for LBP.

Given the observed impact of labels on management intentions, we think clinicians should check patients' understanding of labels and their perceptions of what the labels mean for their individual prognosis and management. For example, patients labelled with *degeneration* may need reassurance that they do not have a serious condition to reduce any psychological distress or uncertainty. Similarly, patients labelled with a *disc bulge* may need reassurance that bulges rarely require intervention and are common in asymptomatic people.

Given the labels *episode of back pain*, *lumbar sprain* and *non‐specific LBP* describe the same clinical presentation and should receive the same management, clinicians can choose which label will be of most value to a patient based on their context and concerns. Findings from this study can inform clinicians' label selection through improved knowledge of the relative risks and benefits. There is a view amongst clinicians that *non‐specific LBP* is an illegitimate diagnosis, that it is unacceptable to patients, that it indicates inadequate clinician expertise to diagnose their problem, and that it may result in seeking further tests and medical opinions ((Bishop et al., 2015; Kent & Keating, 2004). Although there are isolated examples of studies that have reported an ability to diagnose the specific structural cause of most LBP, a systematic review of 41 diagnostic studies found that the prevalence of diagnosable structural causes (e.g. disc, facet joint, SIJ) in people with LBP varied widely and could not be reliably identified using current clinical tests (Hancock et al., 2007). Despite a slight increase in the perceived need for a second opinion compared to *lumbar sprain*, *non‐specific LBP* resulted in the lowest perceived seriousness or need for surgery and *episode of back pain* resulted in the lowest perceived need for imaging. Given there did not appear to be strong differences between the labels *non‐specific LBP* and *episode of back pain*, it could be reasonable—in light of common criticisms of the non‐specific label—to use the latter term. However, the word ‘episode’ denotes something short‐term/acute. Whilst this could encourage positive recovery expectations in people with new back pain, the label ‘*episode of back pain’* may potentially be less acceptable to those who have not recovered—i.e. individuals living with persistent LBP. In our experiment, the label non‐specific LBP was accompanied by the words ‘tension, soreness, stiffness’. These words may help clinicians provide more meaningful/relatable explanations to patients—instead of providing the ‘non‐specific’ label in isolation. *Lumbar sprain* resulted in the most optimistic views of recovery, but may be less relevant for the one‐third of LBP patients who cannot recall an incident that triggered the episode of LBP (Parreira et al., 2015) and would seem more suited to acute than persistent cases of LBP.

### 4.5 Future research

Diagnostic labels are used for many purposes within health systems and broader society. Impacts on these systems and how these support recovery from LBP would need to be considered before any significant re‐labelling was embarked on. In particular, research is required to explore broader system (e.g. insurance companies, workplaces, compensation systems) acceptability of the labels found to be associated with better recovery and reduced need for medical interventions in this study. Individuals with LBP are often (unhelpfully) required to get a structural diagnosis—often through the use of imaging—for their pain to be validated and legitimized (Bartys et al., 2017; Buchbinder et al., 2011). Further, people with LBP are frequently stigmatized and excluded by others, especially when there is no easily communicated underlying medical pathology (Karos et al., 2018). This could present an obstacle to the uptake of the labels *episode of back pain* and *non‐specific LBP* that do not provide a structural diagnosis for an individual's LBP. Several studies suggest that patients want a specific diagnosis that explains their LBP (Lim et al., 2019; Sharma et al., 2020; Verbeek et al., 2004). More research will be required to explore how clinicians can best communicate a symptom in a way that is devoid as possible of words reflecting structural disruption, whilst also meeting patient needs. Broader civic, consumer and clinician involvement in research will be required to examine the desire for updating diagnostic labels and how labels associated with good recovery can be communicated across various contexts.

## 5 CONCLUSION

*Episode of back pain, lumbar sprain* and *non‐specific LBP* reduced the perceived need for imaging, surgery and second opinion compared *to disc bulge, arthritis* and *degeneration* amongst public and patients with LBP, as well as reducing the perceived seriousness of LBP and enhancing recovery expectations. The impact of labels appears most relevant amongst those at risk of poor outcome (participants with current LBP who had a history of seeking care). Little to no difference was found in beliefs about physical activity and work being harmful between the six labels. Clinicians should consider not using the labels *disc bulge*, *degeneration* and *arthritis* as part of explanations and reassurance provided to people with non‐specific LBP. Changing how we label LBP may help reduce unnecessary medical tests and treatments and increase the acceptability of watchful waiting, self‐care and the less intensive treatment options that are recommended in guidelines for the management of non‐specific LBP.

## AUTHOR CONTRIBUTIONS

MOK conceived the idea for the study. All authors contributed to the design of the study. MOK completed the data collection and analysis. MOK drafted the manuscript. All authors contributed to the interpretation of the analysis, and critically revised and approved the manuscript. MOK has full access to all of the data in the study and takes responsibility for the integrity of the data and the accuracy of the data analysis.

## CONFLICT OF INTEREST

None to declare.

## PATIENT AND PUBLIC INVOLVEMENT

One author (JB) is a consumer with persistent non‐specific LBP and provided input at all stages of the study. JB is Co‐chair of the International Association for the Study of Pain Global Alliance of Pain Patient Advocates task force. JB provided substantial advice on the specific labels to be tested at the planning stage, the scenario and label descriptions and provided input on the outcome measures used. At the end of the study, JB commented on the findings and contributed to the dissemination plan.

## Supporting information


Data S1
