# Supplementary material for: Effect of diagnostic labelling on management intentions for non‐specific low back pain: A randomized scenario‐based experiment
Source: Eur J Pain. 2022 Jun 21;26(7):1532–45. doi: 10.1002/ejp.1981 (PMC9545091; doi:10.1002/ejp.1981)
Supplement: Supplementary file 1 — Data S1 [file EJP-26-1532-s001.docx]

**Online Supplement**

EMethods: Study questionnaires

ETable 1: Median (IQRs) for all outcomes

EFigure 1: Mean differences (99.67% CIs) for beliefs about needing a second opinion on a 11 point likert scale

EFigure 2: Mean differences (99.67% CIs) for perceived seriousness of low back pain on a 11 point likert scale

EFigure 3: Mean differences (99.67% CIs) for recovery expectations on a 11 point likert scale

EFigure 4: Mean differences (99.67% CIs) for “Physical might harm my back” on a seven point likert scale

EFigure 5: Mean differences (99.67% CIs) for **“I should not do physical activities which (might) make my back” on a seven point likert scale**

EFigure 6: Mean differences (99.67% CIs) for “Work might harm my back” on a seven point likert scale

EFigure 7: Mean differences (99.67% CIs) for “**I should not do my normal work with my present pain”** on a seven point likert scale

**EMethods:** Study questionnaires

**Questions about management**

1. Based on what your healthcare provider has told you, do you think you need a scan (for example, an X-ray or MRI scan) of your back? 0=Definitely do not 10=Definitely do
2. Based on what your healthcare provider has told you, do you think you need a second opinion from a specialist (e.g. back surgeon) for your low back pain? 0= Definitely do not 10=Definitely do
3. Based on what your healthcare provider has told you, how willing would you be to have surgery for your low back pain if you were advised to? 0=Definitely not willing 10=Definitely willing

**Questions about seriousness and recovery**

1. Based on what your healthcare provider has told you, do you think that being diagnosed with [insert label here] is a serious condition for you to have? 0=Definitely not serious 10=Definitely serious
2. Based on what your healthcare provider has told you, how likely do you think it is that your low back pain will be fully recovered in 3 months?" 0 = Not at all likely 10 = very likely).

**Questions about physical activity**

Based on what your healthcare provider has told you, please choose any number from 0 to 6 to say how much physical activity may affect your back.

1. Physical activity (for example, bending, lifting, walking, exercise, driving) might harm my back? 0=Completely disagree 6=Completely disagree
2. I should not do physical activities which (might) make my back worse 0=Completely disagree 6=Completely disagree

**Questions about work**

Based on what your healthcare provider has told you, please choose any number from 0 to 6 to say how much work may affect your back.

1. My work might harm my back ? 0=Completely disagree 6=Completely disagree
2. I should not do my normal work with my present pain 0=Completely disagree 6=Completely disagree

**Information about you**

**What is your age?** _________

**What is your gender?**

- Male
- Female
- Prefer not to say

**What is the highest level of education you have completed?** (Check only **one** box)

- Primary school or less
- High school (not completed)
- High school (completed)
- TAFE/Trade
- University- Undergraduate degree/s (completed)
- University- Postgraduate degree/s e.g., Master, PhD (completed)

**What is your current employment status?** (Check only **one** box)

- Working full time
- Working part time
- Home duties
- Full-time student
- Part-time student
- Retired
- Permanently unable to work/ill
- Other (please specify)

**Have you ever had low back pain in the past?**

- Yes
- No

**Have you previously had a scan (e.g Xray, MRI) for low back pain?**

- Yes
- No

**Have you previously had surgery for low back pain?**

- Yes
- No

**Have you previously taken sick leave due to low back pain?**

- Yes
- No

**Has a healthcare provider previously given you a cause/name/label for the cause of your low back pain?**

- Yes (please specify) _________________________
- No

**How much low back pain have you had during the past week?**

- None
- Very mild
- Mild
- Moderate
- Severe
- Very severe

**During the past week, how much did low back pain interfere with your normal work (including both work outside the home and housework)?**

- Not at all
- A little bit
- Moderately
- Quite a bit
- Extremely
- Not applicable. I do not have low back pain.

**How long have you had your current lower back pain?**

- 1-2 weeks
- 3-4 weeks
- 4-5 weeks
- 6-8 weeks
- 9-11 weeks
- 3-6 months
- 6-9 months
- 9-12 months
- Over one year
- Not applicable. I do not have low back pain.

**How tense or anxious have you felt in the past week?** 0=not at all. 10=Extremely

**How much have you been bothered by feeling depressed in the past week?** 0= not at all. 10=Extremely

**ETable 1:** Median (IQRs) for each group for all outcomes

|  | **Disc bulge** | **Degeneration** | **Arthritis** | **Lumbar sprain** | **Non-specific LBP** | **Episode of back pain** |
| --- | --- | --- | --- | --- | --- | --- |
| **Median (IQR)** | | | | | | |
| **Primary outcome** | | | | | | |
| Imaging (0-10)^1^ | 6 (3-8) | 6 (3-8) | 6 (4-8) | 4 (2-6) | 4 (2-7) | 4 (2-6) |
| **Secondary outcomes** | | | | | | |
| Second opinion (0-10) | 5 (3-7) | 6 (4-8) | 6 (3-8) | 3 (2-5) | 4 (2-7) | 5 (2-7) |
| Willing to undergo surgery (0-10)^2^ | 4 (2-7) | 5 (2-7) | 4 (2-6) | 3 (1-5.5) | 3 (1-5) | 3 (1-6) |
| Perceived seriousness (0-10) | 6 (5-7) | 7 (5-8) | 6.5 (5-8) | 4 (2-6) | 4 (2-6) | 5 (2-7) |
| Recovery expectations (0-10) | 6 (4-7) | 5 (3-7) | 5 (2.5-6) | 7 (5-8) | 6 (4-8) | 6 (4-8) |
| Physical activity may harm my back (0-6) | 4 (3-5) | 4 (3-5) | 3.5 (3-4) | 3 (2-4) | 3 (3-4) | 4 (2-4) |
| I should not do physical activities … (0-6) | 4 (2-5) | 3 (2-5) | 3 (2-4.5) | 4 (2-5) | 3 (2-5) | 4 (2-5) |
| My work might harm my back (0-6) | 3 (2-4) | 3 (2-4) | 3 (2-4) | 3 (2-4) | 3 (2-4) | 3 (2-4) |
| I should not do my normal work (0-6) | 3 (2-4) | 3 (2-5) | 3 (2-4) | 3 (2-4) | 3 (1-4) | 3 (2-4) |

^1^Adjusted for previous lumbar imaging ^2^Adjusted for previous surgery for LBP

**eFigure 1:** Mean differences (99.67% CIs) for beliefs about needing a second opinion on a 11 point likert scale


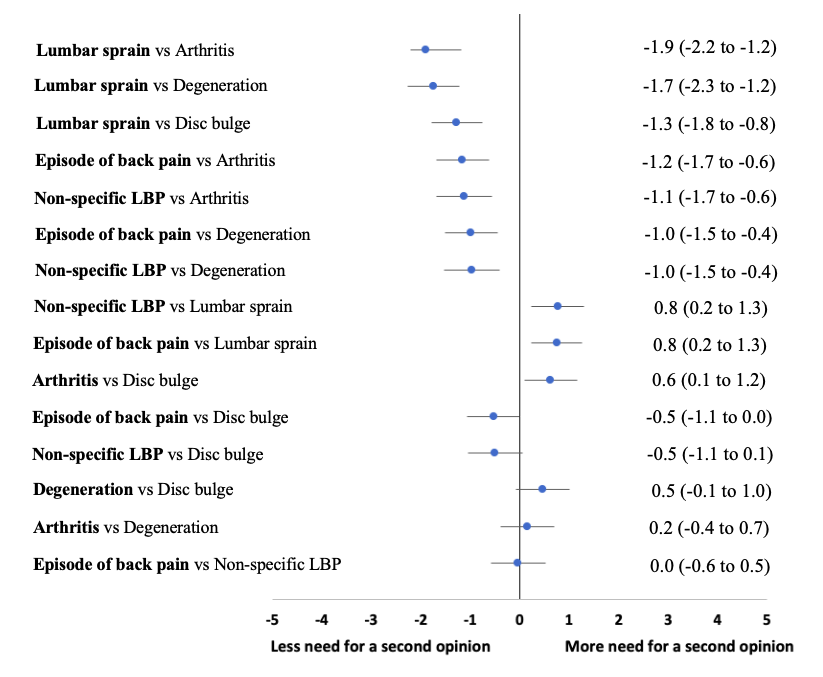


********Experimental label highlighted in* ***bold***

**EFigure 2:** Mean differences (99.67% CIs) for perceived seriousness of low back pain on a 11 point likert scale

***
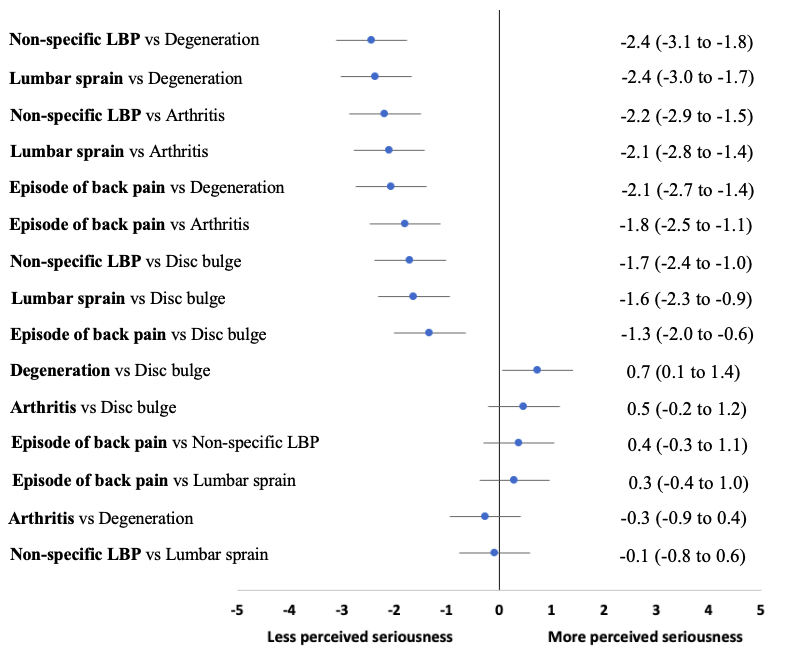
***

********Experimental label highlighted in* ***bold***

**eFigure 3:** Mean differences (99.67% CIs) for recovery expectations on a 11 point likert scale

***
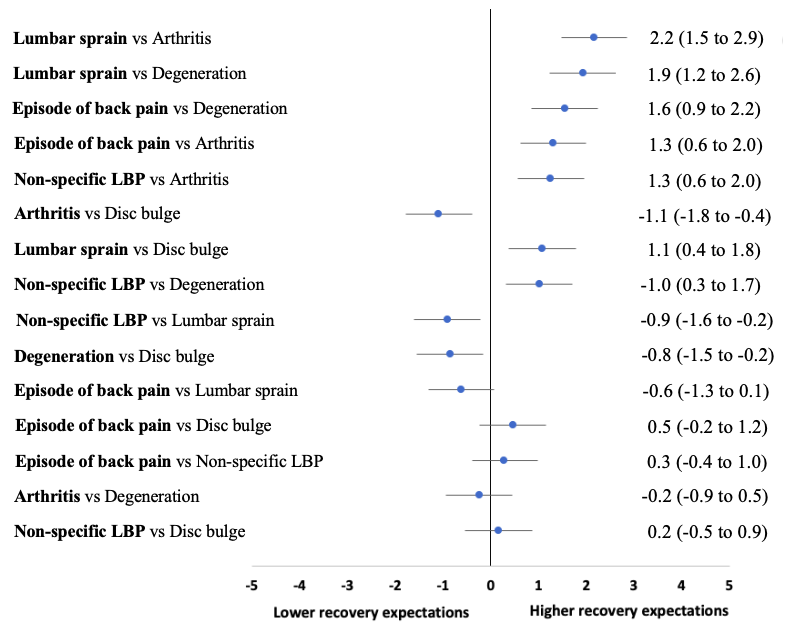
***

********Experimental label highlighted in* ***bold***

**eFigure 4:** Mean differences (99.67% CIs) for “Physical might harm my back” on a seven point likert scale

**
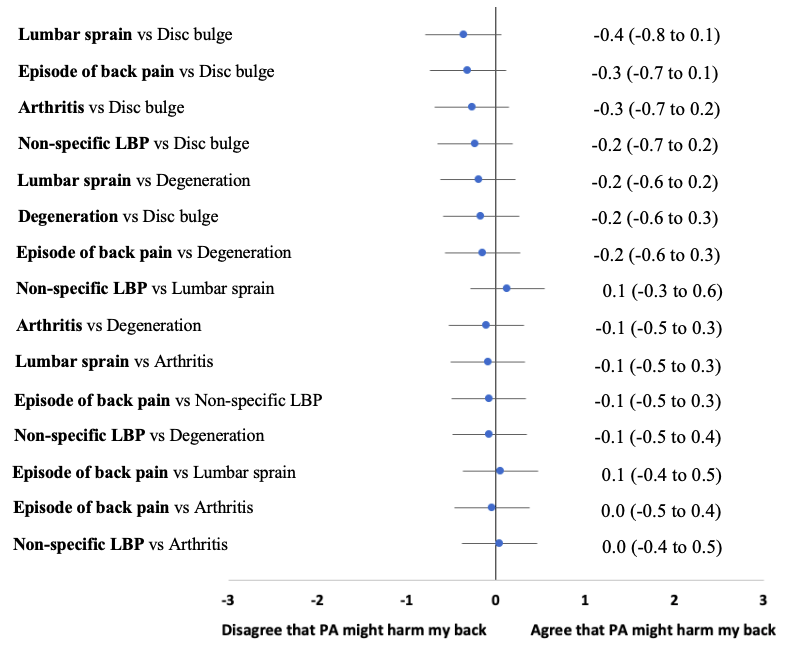
**

********Experimental label highlighted in* ***bold***

**eFigure 5:** Mean differences (99.67% CIs) for “**I should not do physical activities which (might) make my back” on a seven point likert scale**

**
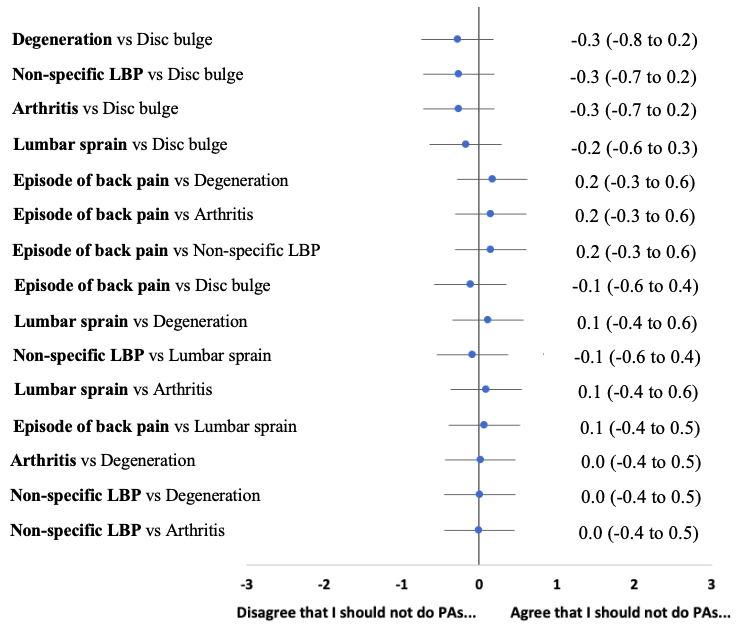
**

********Experimental label highlighted in* ***bold***

**PAs – physical activities**

**eFigure 6:** Mean differences (99.67% CIs) for “Work might harm my back” on a seven point likert scale


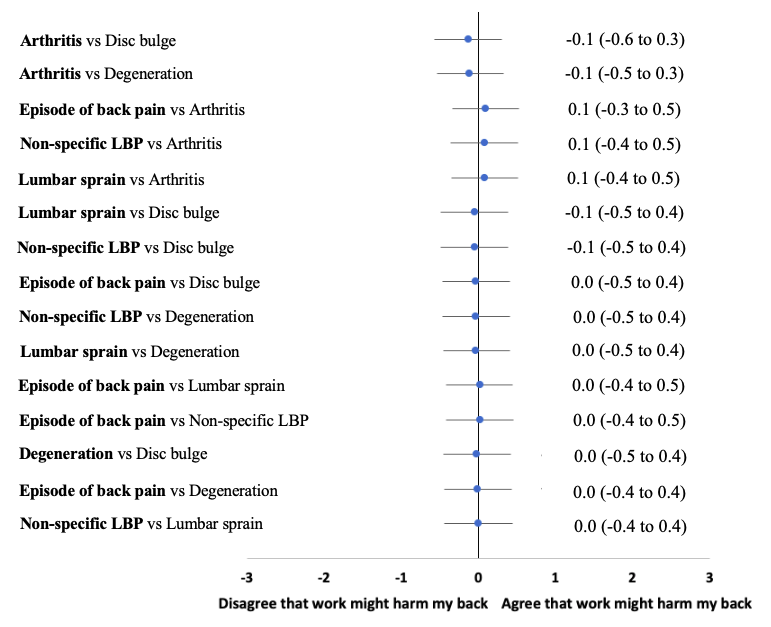


********Experimental label highlighted in* ***bold***

**eFigure 7:** Mean differences (99.67% CIs) for “**I should not do my normal work with my present pain”** on a seven point likert scale


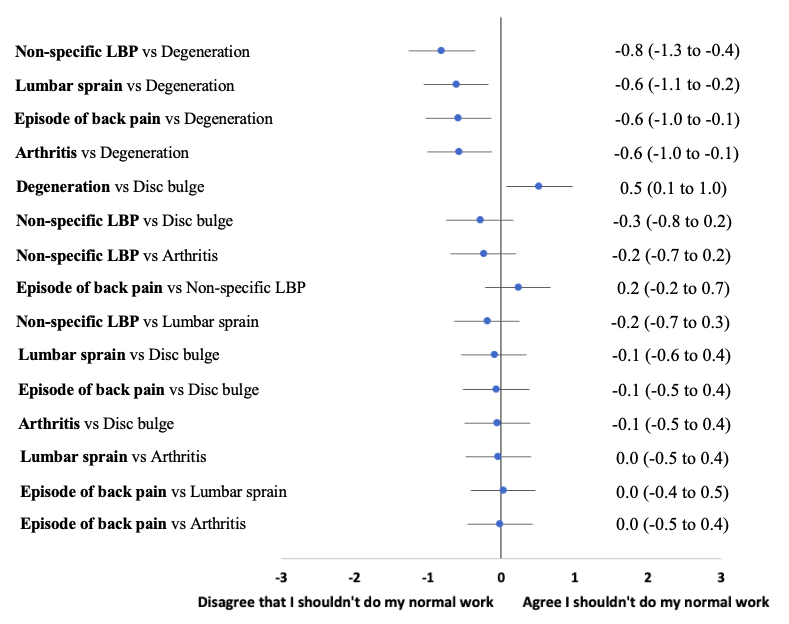


********Experimental label highlighted in* ***bold***
